# Connecting Chassis Metabolism with Glycosyltransferase Catalysis: The Role of Nucleotide Sugars in Human Milk Oligosaccharide Biosynthesis

**DOI:** 10.3390/foods15213851

**Published:** 2026-10-28

**Authors:** Zixuan Chu, Feiyu Zhou, Miaomiao Hu

**Affiliations:** Department of Food Science and Engineering, College of Food Science and Engineering, Jilin University, Changchun 130062, China; zhuzx26@mails.jlu.edu.cn (Z.C.); zhoufy9925@mails.jlu.edu.cn (F.Z.)

**Keywords:** human milk oligosaccharides, nucleotide sugars, chassis metabolism, glycosyltransferases, metabolic allocation, supply–demand coordination

## Abstract

Nucleotide sugars connect host metabolism to glycosyltransferase(GT)-catalyzed human milk oligosaccharide (HMO) biosynthesis, yet larger donor pools do not necessarily increase product formation. This review examines GDP-fucose, UDP-*N*-acetylglucosamine, UDP-galactose, and CMP-*N*-acetylneuraminic acid in relation to precursor competition, nucleotide regeneration, catalytic performance, and host physiology. It distinguishes direct donor consumption from upstream precursor, energy, biomass, and maintenance requirements and compares fermentation, whole-cell conversion, and cell-free biocatalysis. Published studies show that production depends on coordination among donor formation, active GT capacity, acceptor accessibility, competing metabolism, and process conditions. Sampling and extraction artifacts can distort donor-pool estimates, while in vitro kinetic parameters may not predict performance in cellular systems. Manufacturing assessment also requires attention to catalyst lifetime, product recovery, structural quality, and scale-up. An experimental framework is proposed to guide measurements and independent perturbations of donor supply, GT activity, acceptor access, and transport. HMO production should be assessed by yield, productivity, selectivity, structural identity, and stability, rather than donor abundance or endpoint titer alone.

## 1. Introduction

The structures of human milk oligosaccharides (HMOs) are defined by their monosaccharide composition, glycosidic linkages, and the order of glycan extension. Their preparation supports both the development of functional food ingredients and investigations of structure–function relationships. Network-based integration of glycan and gene-expression data has helped identify candidate glycosyltransferases (GTs) involved in mammary HMO biosynthesis [1]. Translation of this biological knowledge into engineered production systems requires separate evaluation of catalytic specificity and host resources. A review focused on lacto-*N*-tetraose (LNT) has outlined its physiological functions and compared chemical, enzymatic, and cell factory production routes [2]. Rational metabolic pathway design has enabled efficient biosynthesis of complex fucosylated HMOs such as lacto-*N*-fucopentaose I (LNFP I) in engineered *Escherichia coli *(*E. coli*) [3]. Metabolic engineering strategies have further been adapted to probiotic *E. coli* Nissle 1917 for the production of core HMO lacto-*N*-triose II (LNT II) [4]. In addition, conceptual frameworks like the pipeline metabolic model have been proposed to guide pathway design and optimization in food synthetic biology [5]. However, a more detailed mechanistic understanding is needed of how these interventions interact through nucleotide sugars in specific production systems.

Traditional strain engineering often increases pathway gene dosage or removes competing reactions and then evaluates the final HMO titer. Such interventions can be useful, but they change several resource demands simultaneously: stronger donor synthesis may consume nucleotides or precursors needed for growth, while additional GT protein may remain inactive or encounter insufficient acceptor. A positive titer response therefore demonstrates an engineering outcome without necessarily identifying the controlling reaction. The modular neutral-core HMO platform [6] illustrates the practical value of coordinating donor supply, catalysis, and transport; it does not establish that any single module controls flux in all production phases. The specific gap addressed here is how to discriminate these explanations before selecting the next intervention.

High enzyme expression accompanied by limited product formation is insufficient, by itself, to establish donor limitation. Conversely, donor accumulation does not demonstrate an increase in productive capacity. A recent study in *E. coli* MG1655 still required coordinated adjustments to GT expression, donor formation, and transport to establish a platform for producing different neutral core HMOs [6]. These findings position nucleotide sugars as the connection between host resource allocation and glycan assembly. As the number of reaction steps increases, endpoint titers become less informative about the relationships among donor formation, acceptor accessibility, and cellular physiology.

**Figure 1:**
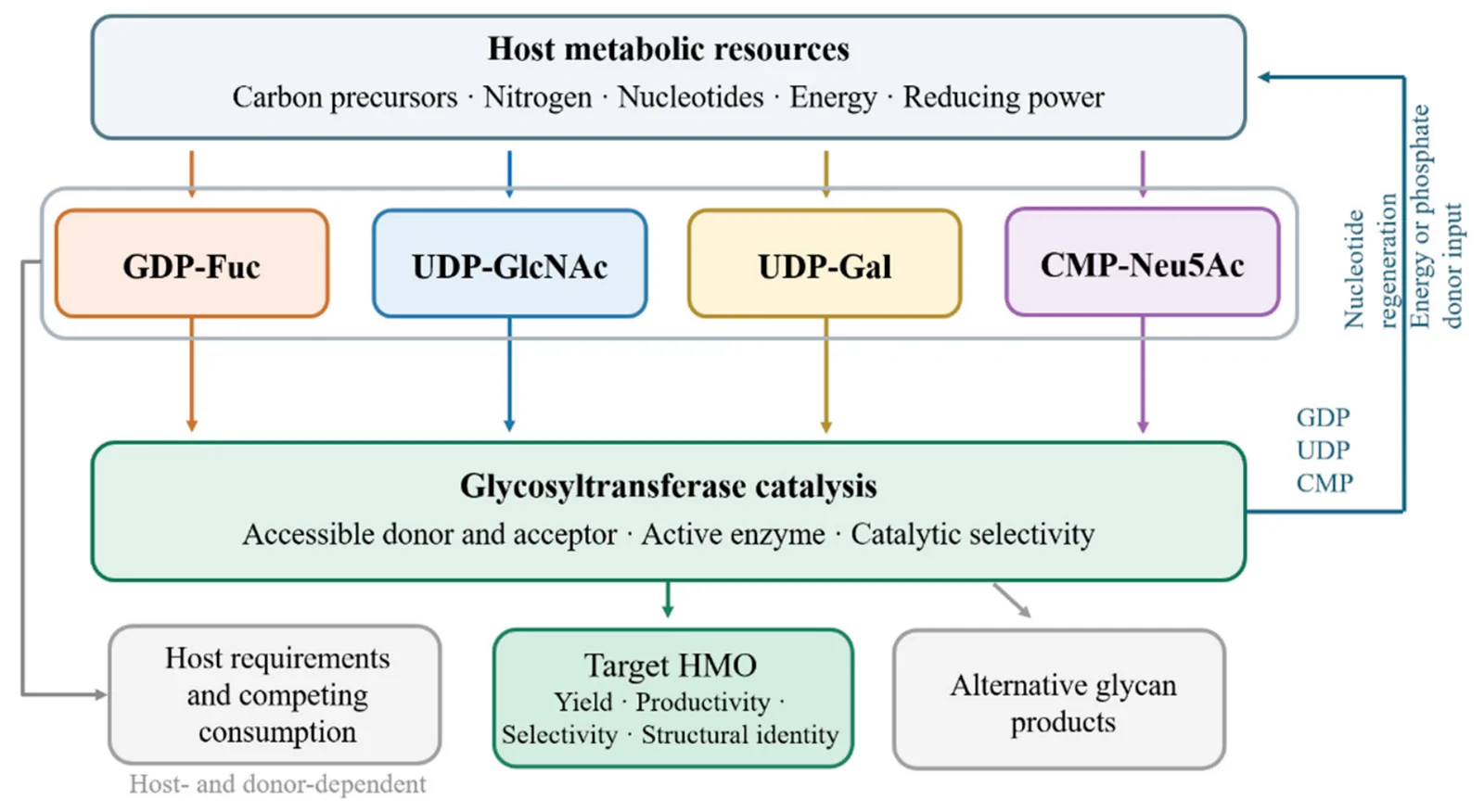
Conceptual framework linking nucleotide sugars to chassis metabolism, glycosyltransferase catalysis, and product formation. The four donors are alternatives or combinations; each HMO does not necessarily consume all four. Target glycosyl transfer, host requirements, and other consumption determine donor turnover. Nucleotides released after transfer can be regenerated and reused for donor activation. Gray branches depend on the host and reaction network. Arrows indicate conceptual relationships, not quantitative fluxes. GDP-Fuc, guanosine diphosphate fucose; UDP-GlcNAc, uridine diphosphate N-acetylglucosamine; UDP-Gal, uridine diphosphate galactose; CMP-Neu5Ac, cytidine monophosphate N-acetylneuraminic acid; GDP, guanosine diphosphate; UDP, uridine diphosphate; CMP, cytidine monophosphate; HMO, human milk oligosaccharide. Regeneration requires an external or metabolically supplied energy input; it is not an energy-neutral loop.

This review examines how nucleotide-sugar formation and regeneration interact with GT activity, acceptor access, competing metabolism, and host physiology during HMO biosynthesis. Microbial fermentation is the primary focus; whole-cell conversion and cell-free biocatalysis are considered for comparison. Donor pool refers to the measured amount of a nucleotide sugar at a defined time, whereas donor availability is the fraction accessible to the relevant enzyme. Biosynthetic flux is material conversion per unit time, and a pool measurement alone cannot establish flux. Figure 1 summarizes these relationships.

## 2. Metabolic Origins and Resource Requirements of Nucleotide Sugars

### 2.1. GDP-Fucose Requires Coordinated Supplies of Carbon Precursors, GTP, and Reducing Power

GDP-fucose (GDP-Fuc) is the direct donor for fucosyltransferase reactions. In the canonical de novo pathway, ManA and ManB convert fructose-6-phosphate to mannose-1-phosphate via mannose-6-phosphate. ManC then uses GTP to generate GDP-mannose, which is converted to GDP-Fuc by Gmd and WcaG. The pathway therefore requires carbon precursors and nucleotides, together with reducing power. Increasing the abundance of the terminal donor-forming enzymes changes only part of the pathway capacity; actual flux remains dependent on upstream carbon allocation, GTP availability, and cofactor status. Studies of 2′-fucosyllactose (2′-FL) production have increased titers through combined modifications to precursor pathways and cofactor-related metabolism, illustrating the coupling among these resources [7,8].

The salvage pathway uses exogenous fucose through fucokinase/GDP-fucose pyrophosphorylase activity and also requires ATP and GTP [9]. Choi et al. reinforced GTP-related metabolism during 3-fucosyllactose (3-FL) production, whereas Deng et al. combined improved substrate transport with GTP regeneration in *Bacillus subtilis* (*B. subtilis*) [10,11]. Both studies illustrate the need to evaluate sugar availability together with nucleotide resources. However, the final titers obtained after combined modifications cannot be attributed entirely to increased GTP supply. Comparisons between the two GDP-Fuc routes should account for exogenous fucose input, the burden of intracellular donor formation, and by-product generation. Pathway length alone is not a sufficient basis for judging process economics.

### 2.2. UDP-N-Acetylglucosamine Is Shared Between Product Formation and Cell Structure

UDP-*N*-acetylglucosamine (UDP-GlcNAc) supplies the GlcNAc residue used to extend lactose to LNT II. In the canonical *E. coli* pathway, GlmS connects fructose-6-phosphate metabolism with glutamine utilization, GlmM shifts the position of the phosphate group, and GlmU uses acetyl-CoA and UTP for subsequent acetylation and activation. UDP-GlcNAc thus brings together carbon, nitrogen, acetyl groups, and pyrimidine nucleotide supply at one metabolic node. It is also an essential precursor for peptidoglycan synthesis and contributes to lipid A and other envelope components in Gram-negative bacteria. Increased allocation to HMO synthesis must remain compatible with the resources required to maintain cell structure.

This allocation is influenced by endogenous regulatory networks. Khan et al. demonstrated that RapZ senses glucosamine-6-phosphate in *E. coli* and regulates its homeostasis through a network involving small RNAs [12]. Their work provides a mechanistic basis for feedback regulation of an amino sugar precursor. The sensed metabolite is glucosamine-6-phosphate, however, so the findings do not establish RapZ as a direct UDP-GlcNAc sensor. The benefit of relieving a particular regulatory constraint in an engineered strain will also depend on downstream conversion, GT-mediated consumption, and envelope biosynthesis. Regulatory mechanisms should therefore be evaluated in the relevant host. In a *B. subtilis* system producing lacto-*N*-neotetraose (LNnT), combinatorial module experiments supported coordinated adjustment of UDP-GlcNAc and UDP-galactose (UDP-Gal) supply [13].

### 2.3. UDP-Galactose Interconversion Must Be Distinguished from Net Donor Pool Formation

UDP-Gal supplies galactose for the conversion of LNT II to LNT or LNnT. GalU uses glucose-1-phosphate and UTP to form UDP-glucose (UDP-Glc), and GalE catalyzes interconversion between UDP-Glc and UDP-Gal. Exogenous galactose can also enter these metabolic reactions through GalK and GalT. Hu et al. coexpressed galE-galT-galK with the LNT assembly pathway and subsequently modified competing branches to establish an effective LNT production system [14]. These results support the value of restructuring donor-related metabolism, although they do not establish equal or independent contributions from each gene.

GalE changes the capacity for interconversion between two donors; it cannot independently generate the total UDP-sugar pool. Further galE overexpression may therefore offer limited benefits when UTP, glucose-1-phosphate, or upstream activation capacity is limiting. Li et al. increased LNT production by combining pathway optimization with UTP regeneration, providing an example of the contribution of nucleotide resources to neutral core HMO synthesis [15]. Donor availability should also be distinguished from the capacity of the terminal reaction. Residual LNT II may reflect insufficient UDP-Gal supply, limited terminal GT activity, acceptor transport, or enzyme inactivation. An extracellular intermediate alone cannot identify which of these processes is responsible.

### 2.4. CMP-N-Acetylneuraminic Acid Introduces an Activation Requirement Beyond Sialic Acid Formation

CMP-*N*-acetylneuraminic acid (CMP-Neu5Ac) is the direct donor used by sialyltransferases; free Neu5Ac cannot substitute for the activated donor in these reactions. In the canonical NeuC/NeuB/NeuA pathway, hydrolytic epimerization of UDP-GlcNAc first generates N-acetylmannosamine. NeuB then uses this precursor and phosphoenolpyruvate (PEP) to form Neu5Ac, which NeuA activates with CTP. Sialylated HMO production consequently depends on CTP supply and activation capacity as well as Neu5Ac formation and terminal transfer. Routes proceeding through UDP-GlcNAc are also connected to upstream UTP demand.

**Figure 2:**
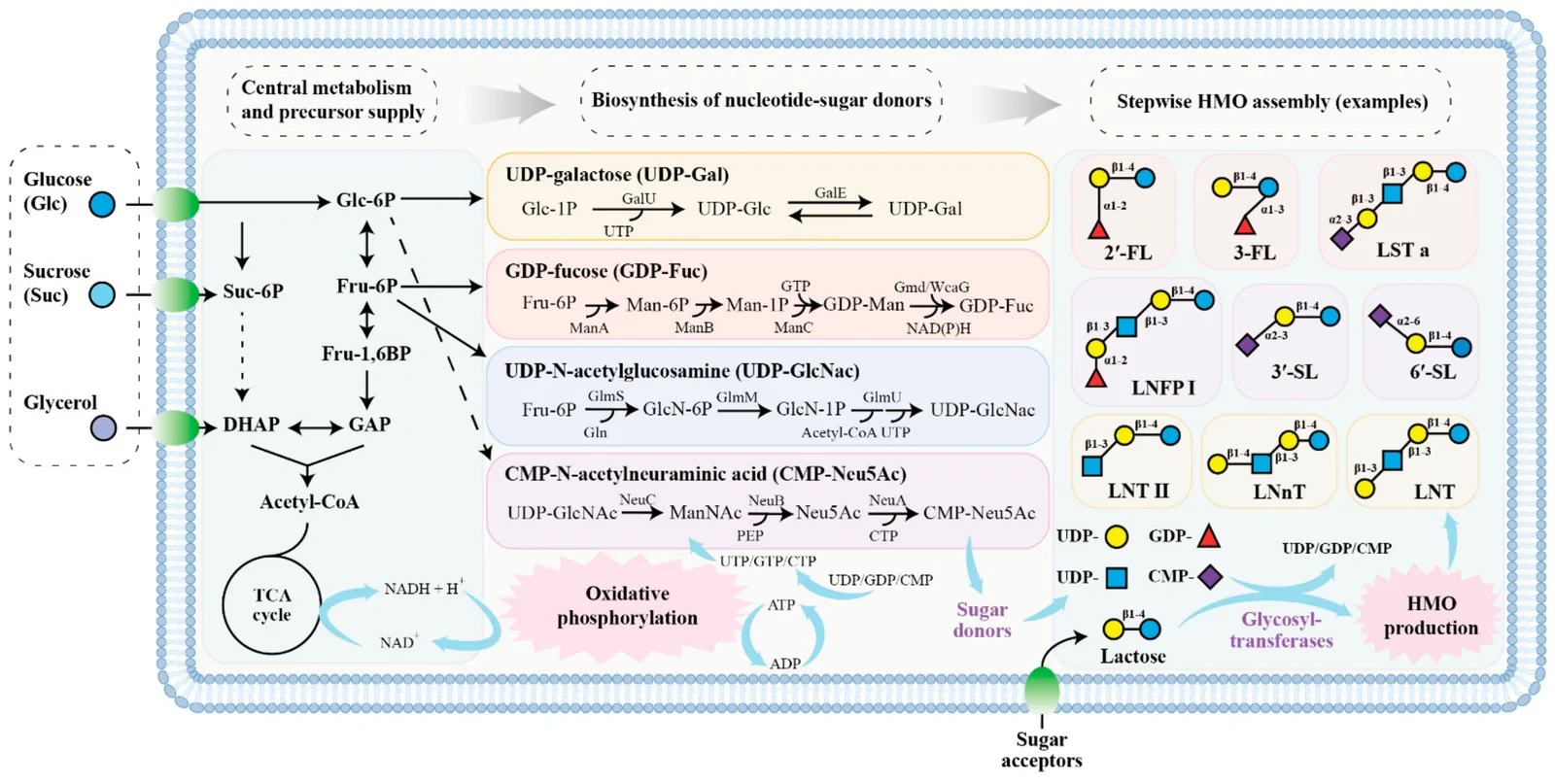
Schematic integration of central metabolism, nucleotide-sugar donor biosynthesis, nucleotide regeneration, and stepwise assembly of representative human milk oligosaccharides (HMOs). Central metabolic intermediates provide carbon precursors, reducing power, nucleotides, and energy for the biosynthesis of UDP-galactose (UDP-Gal), GDP-fucose (GDP-Fuc), UDP-*N*-acetylglucosamine (UDP-GlcNAc), and CMP-*N*-acetylneuraminic acid (CMP-Neu5Ac). UDP-GlcNAc extends lactose to lacto-*N*-triose II (LNT II), which is subsequently galactosylated with UDP-Gal to form lacto-*N*-tetraose (LNT) or lacto-*N*-neotetraose (LNnT). GDP-Fuc supports the formation of fucosylated HMOs, including 2′-fucosyllactose (2′-FL), 3-fucosyllactose (3-FL), and lacto-*N*-fucopentaose I (LNFP I), whereas CMP-Neu5Ac is required for sialylated products such as 3′-sialyllactose (3′-SL), 6′-sialyllactose (6′-SL), and sialyllacto-*N*-tetraose a (LST a). UDP, GDP, and CMP released during glycosyltransferase reactions can be regenerated to the corresponding nucleotide triphosphates through energy- or phosphate-donor-dependent reactions. The diagram combines representative reactions rather than specifying a complete network for every host. Carbon-source entry routes and transport functions are host dependent, and grouped central-metabolic arrows summarize multiple reactions. Abbreviations: Glc, glucose; Suc, sucrose; Gly, glycerol; Fru, fructose; Man, mannose; GlcN, glucosamine; GlcNAc, N-acetylglucosamine; Gal, galactose; Fuc, fucose; ManNAc, N-acetylmannosamine; Neu5Ac, N-acetylneuraminic acid; P, phosphate; BP, bisphosphate; DHAP, dihydroxyacetone phosphate; GAP, glyceraldehyde-3-phosphate; PEP, phosphoenolpyruvate; Gln, glutamine; CoA, coenzyme A; TCA, tricarboxylic acid; ATP/ADP, adenosine tri-/diphosphate; UTP, uridine triphosphate; GTP, guanosine triphosphate; CTP, cytidine triphosphate; NAD(H), oxidized/reduced nicotinamide adenine dinucleotide; NADP(H), its phosphorylated counterpart. Enzymes: GalU, UDP-glucose pyrophosphorylase; GalE, UDP-glucose 4-epimerase; ManA, mannose-6-phosphate isomerase; ManB, phosphomannomutase; ManC, mannose-1-phosphate guanylyltransferase; Gmd, GDP-mannose 4,6-dehydratase; WcaG, GDP-fucose synthase; GlmS, glucosamine-6-phosphate synthase; GlmM, phosphoglucosamine mutase; GlmU, bifunctional acetyltransferase/uridyltransferase; NeuC, UDP-GlcNAc 2-epimerase; NeuB, Neu5Ac synthase; NeuA, CMP-Neu5Ac synthetase. Glycan symbols represent glucose (blue circle), galactose (yellow circle), GlcNAc (blue square), fucose (red triangle), and Neu5Ac (purple diamond).

Lv et al. incorporated CTP regeneration into a 3′-sialyllactose (3′-SL) production system together with pathway and transport engineering, improving overall production [16]. More direct catalytic evidence was provided by Schelch et al., who jointly measured substrates, intermediates, and enzyme activities and used kinetic modeling to coordinate CMP-sialic acid synthetase with sialyltransferase activity. This reduced CMP-Neu5Ac accumulation and improved whole-cell conversion [17]. Enzyme stability also affected the optimal reaction conditions. Excess donor formation relative to terminal utilization can therefore reduce process efficiency, and increasing NeuA activity is not a universal solution. The canonical routes of nucleotide-sugar formation and their coupling to representative HMO assembly are summarized in Figure 2, whereas Table 1 lists the corresponding direct donor stoichiometries.

### 2.5. Direct Stoichiometry and Cellular Resource Costs Have Different Boundaries

For one mole of LNT or LNnT formed from lactose, the direct demand is one mole each of UDP-GlcNAc and UDP-Gal. Using the canonical Glm and GalU/GalE routes, this entails two UTP-dependent activation events, one glutamine-dependent amino-group transfer, and one acetyl-CoA-dependent acetylation. LNFP I adds one GDP-Fuc transfer, one GTP-dependent activation, and a net reducing-equivalent demand in de novo GDP-Fuc formation. The fucose salvage route instead requires fucose phosphorylation by ATP as well as GTP-dependent activation. Each NeuC-route sialyl residue adds an upstream UDP-GlcNAc formation event, one PEP input, and CTP-dependent Neu5Ac activation. Consequently, sialyllacto-*N*-tetraose a (LST a) and sialyllacto-*N*-tetraose c (LST c) have three upstream UTP-dependent activation events plus one CTP-dependent activation event under these particular routes. These are pathway bookkeeping requirements, not measurements of intracellular ATP expenditure; alternative salvage routes change the accounting (Table 1 and Supplementary Table S1).

Recycling UDP or GDP to the corresponding triphosphate requires one phosphorylation step, whereas CMP-to-CTP regeneration requires two; the phosphate donor and enzyme specificity depend on the implemented system. Activation of a sugar-1-phosphate or Neu5Ac by a nucleoside triphosphate releases pyrophosphate, so activation and recycling must be counted together. ATP can support nucleotide phosphorylation and precursor metabolism, but oxygen supply, maintenance requirements, and the regeneration architecture determine the effective energetic burden. Repeated cycling also requires compensation for nucleotide degradation or loss. Overexpressing a donor-forming enzyme cannot restore a depleted nucleotide or phosphate-donor supply by itself [10,11,15,16,17,21,22].

Stoichiometric feasibility does not guarantee a favorable driving force. Reaction direction depends on transformed Gibbs energy under the actual substrate and product activities, pH, ionic conditions, and metal binding; enzymes alter reaction rates but not equilibrium. Pyrophosphate hydrolysis can favor donor activation, but the overall network still depends on nucleotide ratios and energy input. Thermodynamic analyses of central metabolism show that weak driving forces can increase the enzyme demand required to sustain net flux [23]. These analyses motivate similar evaluation of HMO pathways but do not establish their intracellular reaction energies. Numerical driving forces require condition-specific concentrations and reliable thermodynamic data.

Competition is donor- and host-specific. UDP-GlcNAc feeds bacterial envelope formation and, in yeast, chitin and glycosylation; UDP-Glc/UDP-Gal can be distributed to envelope glycans, extracellular polysaccharides, or other glycosylation pathways when those routes are present. GDP-mannose can support host glycan synthesis, and GDP-Fuc can enter native fucosylated polysaccharides in strains carrying the appropriate pathways. Neu5Ac can be degraded, while CMP-Neu5Ac may be consumed by native sialylation only in hosts with the relevant machinery. These are not universal sinks in every chassis. Excess diversion can manifest as slower growth, envelope defects, altered morphology, or loss of viability; direct competition must be distinguished from expression burden [12,13,24,25,26,27,28,29]. At high cell density, fluctuating oxygen and feed availability can further restrict nucleotide regeneration. Relevant tests pair HMO-specific production rates with viability, leakage, morphology, substrate uptake, and oxygen-response measurements, rather than assuming that deleting an apparently competing pathway is physiologically neutral.

## 3. Coupling Donor Formation to Glycosyltransferase Catalysis

### 3.1. Donor Pool Size Is Not Equivalent to Biosynthetic Flux

Donor abundance is a state variable determined jointly by formation and consumption, whereas flux describes the amount of material converted per unit time. For an intracellular donor pool expressed per unit biomass, its rate of change can be represented conceptually as formation minus consumption by the target transfer reaction, host metabolism, degradation, and growth dilution. This mass balance explains why a small donor pool may indicate inadequate upstream supply or rapid turnover, while a large pool may reflect limited downstream utilization. Absolute quantification of intracellular metabolites can inform analyses of reaction rates and enzyme saturation, but interpretation remains dependent on the experimental conditions [30].

For biomass-normalized pool measurements, growth dilution must be separated from chemical consumption. Even an approximately constant pool can support a high formation and consumption rate; conversely, a rising pool can accompany falling product formation if utilization slows. Thus the sign of the pool change alone cannot identify the controlling reaction. An engineering claim about donor limitation should specify the cultivation phase and be supported by a selective perturbation that increases productive rate under otherwise comparable conditions.

In their LNnT-producing system, Dong et al. found that further reinforcement of the UDP-Gal module did not consistently improve production when the UDP-GlcNAc module was relatively weak; some combinations instead reduced the titer [13]. This supports a condition-dependent requirement for coordination between donors, without establishing an optimal concentration ratio applicable to other hosts. A more informative comparison would track donors, intermediates, products, and biomass over time under matched cultivation conditions, allowing the destination of additional metabolic investment to be assessed.

### 3.2. In Vitro Enzyme Performance Must Be Evaluated Against Intracellular Conditions

Under the simplifying assumption of an abundant acceptor and otherwise fixed conditions, the donor concentration relative to the apparent Michaelis constant (*K*_m_) can indicate the potential benefit of increasing donor availability. Actual GT reactions generally involve two substrates, however, and a single-substrate relationship cannot replace a complete kinetic description. When donor availability is well below effective saturation, increasing its concentration may accelerate the reaction. At higher availability, the amount of active enzyme, acceptor concentration, product inhibition, or enzyme inactivation may become more restrictive. Analyses across biological systems have likewise shown that in vivo catalytic rates and in vitro catalytic constants (*k*_cat_) cannot be treated as interchangeable without accounting for their context [31].

The apparent *K*_m_ indicates half-maximal rate only under the conditions and kinetic model used to estimate it; it is not generally a binding affinity. Calculation of *k*_cat_ requires a defensible estimate of active enzyme concentration. At low donor concentration with nonlimiting acceptor, *k*_cat_/*K*_m_ can help prioritize enzymes; near donor saturation, *k*_cat_ and the amount of active enzyme become more informative. These comparisons should be made at matched acceptor, pH, temperature, metal-ion, and ionic conditions. A lower *K*_m_ does not guarantee improved in vivo productivity if the variant has lower turnover, poorer acceptor selectivity, or faster inactivation [17,31].

A practical screen varies both donor and acceptor, measures nucleotide-product effects, and tests whether high substrate concentrations cause inhibition. Initial-rate fits should be complemented by progress curves and residual-activity assays. Michaelis–Menten kinetics assumes a suitable quasi-steady state and effectively constant active enzyme over the measurement interval; accumulated products, reversibility, substrate inhibition, or loss of active protein can invalidate a simple irreversible initial-rate model. Macromolecular crowding and compartmentation add uncertainty, but do not by themselves prove that Michaelis–Menten behavior is impossible. Purified enzymes, crude lysates, resting cells, and permeabilized cells require separate validation of these assumptions.

Screening GTs solely by their maximum activity at high donor concentrations may therefore overlook candidates better suited to intracellular conditions. Donor responsiveness, acceptor selectivity, and stability over the relevant reaction timescale should be evaluated together. The effects of enzyme activity ratios and inactivation observed by Schelch et al. illustrate the value of measuring donor-forming and terminal catalytic capacities separately before combining them [17]. The same reasoning explains why enhancing donor supply or enzyme expression may produce different responses at different stages of a process.

A concrete example is the sialylation module studied by Schelch et al. [17]: loss of transferase activity at 37 °C constrained conversion, whereas operation at 25 °C improved the useful reaction window. This demonstrates why a temperature giving a favorable initial rate need not maximize accumulated product. For engineering, improving stability or limiting inhibitory nucleotide accumulation may therefore outperform further donor reinforcement. Proteomics or soluble protein abundance alone cannot substitute for an assay of active catalyst under relevant reaction conditions.

GDP, UDP, or CMP released after glycosyl transfer reconnects the consumption side of the pathway to nucleotide regeneration. Regeneration requires energy or phosphate donors and must be coordinated with the next round of sugar activation. Donor cycling should therefore be assessed from actual turnover and target product formation, rather than inferred from the expression of a regeneration enzyme. In vitro platforms for UDP-sugar and GDP-Fuc synthesis provide examples in which these connections can be adjusted independently [21,22].

### 3.3. Multiple Donor Requirements Interact with Reaction Selectivity in Complex HMOs

The canonical routes extending lactose to LNT and LNnT directly consume both UDP-GlcNAc and UDP-Gal. LNFP I additionally requires GDP-Fuc, whereas LST a and LST c introduce a requirement for CMP-Neu5Ac. These products have been obtained through combined optimization of donor pathways and multiple GTs [18,19,20]. Direct reaction stoichiometry nevertheless captures only part of the resource demand in a living cell. For example, formation of CMP-Neu5Ac through the NeuC route indirectly consumes UDP-GlcNAc upstream, creating competition for a precursor shared by different pathways.

Donor regulation changes relative reaction rates within an existing catalytic network but does not replace enzyme specificity for a glycosidic linkage. Providing more UDP-Gal will not convert a β1,3-specific GT into a β1,4-specific enzyme. Conversely, an enzyme with broader acceptor compatibility may extend the intended product further, so increased donor availability may also promote longer-chain by-products. Tao et al. modified the acceptor-binding region of LgtA and reduced the proportion of long-chain LNnT derivatives, showing that structural purity also depends on discrimination between target and alternative acceptors [32]. Engineering pathways with multiple donors should therefore include product profiling alongside target quantification. Figure 1 summarizes the connections among donor formation, competing consumption, and catalytic utilization.

## 4. Identifying Nucleotide Sugar Limitations and Interpreting the Evidence

### 4.1. Intracellular Donor Analysis Complements Extracellular Sugar Quantification

Extracellular measurements of HMOs and their precursors cannot substitute for intracellular nucleotide sugar analysis. Intracellular measurements require control of continued metabolism after sampling, extraction losses, and matrix effects, together with a clearly stated basis for normalization, such as cell dry weight, cell number, or intracellular volume. Liquid chromatography–mass spectrometry provides a basis for combined nucleotide and nucleotide sugar analysis [33]. However, isomers such as UDP-Glc and UDP-Gal can share the same mass and similar fragments, making mass-to-charge ratios alone insufficient for identification. A mixed-mode chromatography method developed by Rahm et al. illustrates the importance of resolving these isomers [34]. The method was established in human fibroblasts; transfer to fermentation biomass requires validation of recovery, stability, and the quantitative range.

Sampling artifacts deserve explicit consideration before a low pool is interpreted as donor shortage. Delayed quenching permits ongoing synthesis, consumption, and nucleotide interconversion, so bias can occur in either direction. Leakage during quenching or washing and incomplete extraction can lower apparent intracellular pools, while residual medium can inflate them. Bolten et al. demonstrated substantial leakage under tested cold-methanol protocols in several bacteria [35]; this is evidence for protocol validation, not a universal correction factor for nucleotide sugars. Matrix-dependent ion suppression and selective degradation can further distort comparisons between strains or cultivation phases.

Validation should record sampling-to-quench time, test analyte stability and recovery, and distinguish extracellular medium, quench/wash fractions, and cell extracts when assessing leakage. Matrix-matched calibration and suitable isotope-labeled internal standards should be used where available. Standards added after extraction can assess instrumental response but cannot reconstruct metabolic changes or leakage before their addition; pre-extraction spikes likewise do not necessarily reproduce the release of endogenous intracellular metabolites. Recovery and leakage should therefore be evaluated separately. A reported low concentration without these controls is an uncertain pool estimate and cannot support a confident flux-limitation diagnosis [33,34,35].

Donor measurements should be accompanied by nucleotide profiling. Measuring UTP, GTP, CTP, and related phosphorylation states can help distinguish limitations in sugar precursors from limitations in activation resources. An increase in a nucleotide pool does not itself demonstrate increased regeneration flux. Changes in the relevant substrates and products are also needed to determine whether that nucleotide supports productive donor turnover. Comparisons between strains particularly require consistent cultivation stages and biomass normalization.

### 4.2. Targeted Perturbations Can Distinguish Competing Explanations

An increase in product formation after donor-pathway reinforcement demonstrates an engineering benefit but does not, by itself, identify donor supply as the limiting step. Changes in gene expression can also affect growth, global metabolism, and the burden of protein production. Mechanistic attribution requires measurements linking donor pools and intermediates to product formation, together with independent perturbations and, where feasible, flux analysis.

For LNT II accumulation, for example, donor-forming capacity and terminal enzyme capacity can be varied separately while monitoring the distribution of intermediates inside and outside the cells. When donor pools increase without a corresponding product increase, acceptor supply, enzyme inactivation, and competing consumption should be examined. Stable isotope tracing can help distinguish donor formation from utilization, as demonstrated in studies of nucleotide sugar metabolism [36]. Labeling fractions alone are not absolute fluxes and must be interpreted together with pool sizes, time courses, and a reaction network model. Findings from mammalian cells also require validation before being applied to HMO-producing microorganisms.

The decision sequence proposed in Table 2 begins with analytical validation, then compares time-resolved product formation and donor/intermediate pools, and finally tests donor synthesis, GT capacity, acceptor access, transport, and competing sinks separately. A donor-by-GT factorial perturbation is particularly useful for detecting coupled limitations. These are discriminating tests rather than deterministic diagnoses: more than one process may respond, and all interventions require growth and expression-burden controls.

The tests are proposed to distinguish candidate limitations; their diagnostic performance has not been prospectively validated. GT, glycosyltransferase. Low/High are relative to matched conditions, not universal thresholds.

### 4.3. Limiting Processes Depend on Time and Operating Conditions

The transition from rapid growth to product accumulation changes cellular requirements for structural components, proteins, and energy. A given donor may therefore face different competing demands over the course of cultivation. Endpoint samples can miss transient imbalances following induction or substrate feeding. Quorum-sensing studies in LNnT- and fucosyllactose-producing systems have demonstrated the engineering value of controlling when pathway expression begins [37,38]. Cell-density responses and direct nucleotide sugar feedback are distinct control mechanisms, however, and the benefits of quorum sensing do not establish real-time donor sensing.

Evaluation of donor engineering should consequently cover growth, the onset of pathway expression, and the production phase, with reassessment after changes in carbon source or feeding conditions. Intracellular donor states should not be assumed to remain constant during transfer from shake flasks to bioreactors. Dynamic measurements are intended to identify when supply and consumption diverge, thereby providing a defined target for subsequent regulation. Table 3 summarizes representative engineering strategies, reported outcomes, and the interpretations supported by those results.

Time-course metabolomics resolves pool trajectories but does not identify flux without a model. Isotope tracing identifies precursor incorporation and turnover; labels in the sugar and nucleotide portions must be distinguished, because whole-molecule enrichment can combine several routes [36]. Quantitative flux inference requires measured uptake/secretion, pool sizes, labeling dynamics, a specified network, and uncertainty analysis. Conventional steady-state flux analysis is inappropriate across a rapid feed or induction transition unless a suitable dynamic model is used. Enzyme assays add catalytic information but can lose intracellular inhibition or localization after extraction. Dynamic perturbations improve causal discrimination but may activate compensatory pathways. Supplementary Table S2 summarizes these complementary measurements and their limits.

Experimental systems and units are retained from the original studies. Most endpoints reflect combined interventions and are not single-factor effects of donor reinforcement. Values are not ranked across systems with different substrates and reaction times.

## 5. Coordinating Metabolism and Catalysis for HMO Manufacture

### 5.1. Host Resources Should Be Allocated According to Donor Requirements

*E. coli*, *B. subtilis*, *Saccharomyces cerevisiae*, and *Corynebacterium glutamicum* have all been used for HMO production, but they differ in endogenous donor pathways, cell structure requirements, and the environment for heterologous expression [24,25,26]. Chassis selection should account for the cost and accessibility of the target donor in each host. In eukaryotic systems, GT localization and donor access to the relevant compartment also require attention. Engineered plant platforms have broadened the range of hosts available for HMO synthesis, while retaining the need to consider compartment-specific donor availability [27].

The engineering objective should be productive catalytic capacity per unit of cellular resource expenditure. Heterologous protein synthesis consumes finite host resources, and studies of cellular capacity and burden-responsive feedback have provided quantitative approaches for measuring and adjusting these demands [28,29]. These approaches offer transferable concepts for HMO systems, although their effects on donor utilization and sustained production still require validation. Interventions in branches serving essential cell structures must accommodate physiological requirements. A short-term change in product formation accompanied by impaired growth is insufficient to establish successful resource reallocation. Table 4 summarizes the host-dependent differences in nucleotide-sugar metabolism, engineering constraints, product transport, and food-production considerations.

**Table 4:** Host-dependent considerations for nucleotide-sugar-dependent HMO production.

Host and Evidence	Donor Pathways and Competition	Engineering Opportunities and Burden	Transport and Food-Production Boundaries
*E. coli* [3,4,6,7,8,9,10,14,15,16]	Native UDP-GlcNAc and UDP-Glc/Gal metabolism; GDP-Fuc and CMP-Neu5Ac capacity is strain- and pathway-dependent	Extensive genome and expression tools; strong fermentation precedent; UDP-GlcNAc competes with envelope synthesis	Lactose uptake and HMO export require separate design; protein burden and lysis must be monitored; host-derived impurities require removal. A probiotic strain designation does not establish ingredient safety.
*B. subtilis* [11,13,24]	UDP-GlcNAc serves peptidoglycan; UDP-sugar supply and heterologous fucosylation modules need balancing	Genome integration and modular pathway engineering demonstrated; growth can respond strongly to GT expression level	Lactose transport may require engineering; secretion ability for proteins does not imply HMO export. Strain traits, spores and residual proteins require process-specific control.
*S. cerevisiae* [25,38]	Native GDP-mannose and UDP-sugar metabolism; heterologous GDP-Fuc/GT functions introduced for fucosyllactose	Established yeast genetics and fermentation; donor diversion intersects with cell-wall/glycosylation demand and expression burden	Lactose access requires engineering; cytosolic donor measurement may not represent organellar access. Food-use history cannot substitute for evaluation of the engineered strain and purified product.
*C. glutamicum* [26]	Engineered GDP-Fuc and fucosyltransferase functions enable 2′-FL formation; native central precursors require allocation	Industrial bacterial chassis with demonstrated 2′-FL feasibility; tool and pathway performance remain construct-specific	Transport and cell-envelope constraints must be tested; single-product feasibility cannot be extrapolated to complex HMO assembly or food qualification.
Engineered plants [27]	Native nucleotide-sugar networks and compartmentalized glycosylation can support diverse HMO structures	Photosynthetic production and structural diversification demonstrated; expression and compartment access differ from microbial processes	Recovery from plant tissue and variable expression complicate comparison with broth titers; this is a complementary platform, not a fermentation-equivalent food manufacturing claim.

The table combines reported production examples with mechanistic design considerations; it is not a ranking or a designation of regulatory approval. Genetic accessibility, competing pathways, and impurity risks must be checked for the actual strain and construct.

### 5.2. Donor-Forming Enzymes and Glycosyltransferases Should Be Adjusted Together

Nucleotide sugar engineering changes the reaction environment experienced by a GT, whereas enzyme engineering changes how the donor is used within that environment. Screening should therefore consider donor-forming enzymes, terminal GTs, and acceptor conversion together. For a multistep pathway, the operating range supported by each module can first be characterized, followed by combinatorial expression to adjust their relative capacities. The neutral core HMO platform reported in 2026 illustrates the feasibility of coordinating donor, GT, and transport modules [6].

Spatial organization may also influence intermediate transfer. Docking domains have shown potential for altering the efficiency of assembled heterologous pathways [39]. Enzyme proximity alone, however, does not establish substrate channeling or demonstrate reduced competition from host nucleotide sugar metabolism. Spatial engineering in HMO systems requires measurements of intermediate distributions and reaction kinetics, together with appropriate controls. Such evidence would be needed to establish local enzyme organization as a strategy for donor allocation.

### 5.3. In Vitro Regeneration Systems Can Resolve Coupling and Extend Manufacturing Options

Cell-free biocatalysis allows enzyme ratios, donor regeneration, and acceptor input to be varied independently, providing a more controlled setting for identifying limiting reactions. Hoang et al. developed a multienzyme platform for UDP-GlcNAc and UDP-Gal synthesis and coupled it to HMO production. Cao et al. combined de novo GDP-Fuc synthesis with regeneration of GTP and reducing cofactors for 2′-FL preparation [21,22]. These studies show that in situ donor formation and recycling can reduce the need to add large amounts of preformed nucleotide sugars. Sugar substrates, phosphate donors, or other energy sources remain necessary, and cycling does not eliminate resource consumption.

Optimal enzyme ratios obtained in vitro may provide a starting point for cellular systems, but fermentation additionally involves protein expression, growth, and competing metabolism. Whole-cell conversion must also distinguish among metabolically active, resting, and permeabilized cells because membrane permeability and energy provision differ. Downstream processing contributes to the overall value of each route. For example, multistep one-pot enzyme reactions combined with removable tags have simplified the isolation of HMO intermediates [40]. Application to food ingredient manufacture requires a process-specific assessment of tag removal, residual materials, enzyme preparation, and feedstock costs; reaction yield alone cannot establish industrial superiority. The resource requirements, advantages, limitations, and application boundaries of different HMO production platforms are summarized in Table 5.

**Table 5:** Distinct operating boundaries of HMO production platforms.

Platform	Resource Boundary	Advantages and Limitations	Application and Cost Boundary
Growing-cell fermentation	Metabolism supplies precursors and energy; biomass and maintenance consume resources	Catalysts can be synthesized and replaced; oxygen transfer, growth–production conflict, and genetic stability constrain scale-up	Best assessed for sustained production from economical feedstocks; include seed cultivation, aeration, substrate losses, cell removal and purification.
Resting intact-cell conversion	Reduced growth demand, but viability, maintenance and substrate transport remain important	Avoids individual enzyme purification; activity decays and membrane transport can constrain conversion	Useful for a short cascade with accessible substrates; include catalyst cultivation, preparation, reuse and recovery, not conversion time alone.
Permeabilized-cell conversion	Improved substrate access, with possible cofactor leakage and loss of endogenous regeneration	Separately optimize catalyst loading, enzyme stability and external energy supply	A mechanistic test or conversion platform; include permeabilization, residual agents, nucleotide input and spent-cell removal [17].
Cell-free synthesis	Defined enzymes or lysates permit direct donor/energy control; no growth during conversion	Transport barriers are removed; enzyme preparation, inactivation, inhibition, salts and cofactor losses remain	Useful for modular screening and structurally complex targets; economic suitability depends on catalyst lifetime, recycling, product concentration and purification [21,22,40].

A quantitative economic comparison should use cost per mass of isolated, specification-compliant HMO, rather than reaction yield alone. The numerator includes feedstocks, catalyst preparation and replacement, nucleotide/cofactor makeup, energy or phosphate donors, utilities, waste handling, and downstream processing; the denominator is target mass after recovery and quality rejection. In a prospective sensitivity calculation, increasing downstream recovery from 50% to 80% at unchanged upstream cost reduces the upstream cost contribution per isolated kilogram to 0.50/0.80 = 62.5% of its original value. This is an accounting illustration, not an observed HMO process saving; extra purification expenditure may offset it. Catalyst cost must likewise be divided by cumulative acceptable product across reuse cycles, with measured activity loss rather than an assumed constant lifetime (Supplementary Table S3).

Published demonstrations illustrate the boundary problem. The 13.18 mM 2′-FL endpoint in the cell-free cascade [22] corresponds to approximately 6.44 g/L on an anhydrous molecular-mass basis (488.44 g/mol), calculated only to clarify concentration units. It is not an isolated yield or a profitability estimate. Likewise, the 40 ± 4 g/L 3′-SL whole-cell result [17] used externally supplied CTP in the characterized conversion; its reported reaction time excludes catalyst preparation. These results cannot be ranked economically against fermentation titers. Lysate-based enzyme supply [21] may reduce purification requirements during catalyst preparation, but residual host materials and downstream product purification remain. Public laboratory endpoints without matched catalyst, regeneration, recovery, and scale-up data do not establish which platform has the lower manufacturing cost.

### 5.4. Donor Utilization Should Be Evaluated Alongside Product Quality and Production Stability

HMO production performance should include titer, yield, productivity, and product selectivity. Studies using exogenous fucose, Neu5Ac, or different carbon sources have different substrate input boundaries, so endpoint titers are unsuitable for direct ranking. For pathways requiring multiple donors, the fates of intermediates and by-products should also be reported where possible, allowing the benefit of enhanced supply to be related to the additional metabolic investment. A higher target product fraction may reduce separation requirements, but the economic benefit needs to be supported by process data.

Production stability requires evaluation over sufficiently long cultivation periods and repeated batches. A single high-titer result cannot establish sustained coordination between donor supply and enzyme activity or substitute for an assessment of genetic stability. Structural identity and isomer composition are particularly relevant to food applications; retention time or a single mass signal alone is insufficient for complete structural assignment. The endpoint for nucleotide sugar engineering should be reproducible production of a defined structure and quality.

Structural quality should be assessed using separation of relevant isomers and orthogonal evidence, such as comparison with authentic standards, diagnostic enzymatic digestion, validated tandem mass spectrometry, and nuclear magnetic resonance where required for linkage assignment. Molecular mass alone cannot distinguish LNT from LNnT or 3′-SL from 6′-SL. Report target titer together with molar selectivity, residual lactose, pathway intermediates, longer-chain products, and isolated recovery; these determine both usable product and separation difficulty [32,40].

Scale-up assessment should explicitly test genetic retention and production without continuous selective pressure where applicable, enzyme or cell lifetime, feed and oxygen heterogeneity, and batch-to-batch consistency. Food-ingredient suitability is a product- and process-specific evaluation: potential host-derived proteins, nucleic acids, cell residues, and process reagents require appropriate control. A host with a history of food use, a high analytical purity value, or a high titer does not by itself establish that a new HMO preparation meets the requirements of its intended market. Regulatory status and permitted uses must be checked for the specific ingredient and manufacturing process at submission or commercialization; no blanket approval of hosts or HMOs is implied here.

## 6. Conclusions and Outlook

Nucleotide sugars convert the carbon, nitrogen, nucleotide, and energy resources of the chassis into activated donors that GTs can use. They provide an essential connection for understanding the efficiency of HMO biosynthesis. Existing studies demonstrate the engineering value of donor pathway reinforcement, nucleotide regeneration, and coordinated enzyme expression, while also showing that their effects depend on the host, target product, and operating conditions. Increased donor concentrations do not necessarily indicate increased productive flux, and the direct donor stoichiometry of complex HMOs does not fully describe cellular resource requirements.

Further work should prioritize reliable dynamic measurements and independent perturbations of donor formation, competing consumption, and terminal utilization. In pathways involving multiple donors and assembly steps, acceptor selectivity, expression burden, and reaction timing should be optimized alongside donor supply. Linking these measurements to yield, selectivity, and production stability may support more transferable principles for nucleotide-sugar engineering and improve control of the relationship between metabolism and catalysis in HMO manufacture. The proposed decision framework remains to be tested across hosts and cultivation phases. Comparisons are currently constrained by differences in process boundaries and limited paired measurements of intracellular flux and industrial cost. Available evidence therefore does not support a universal donor-pool threshold, optimal donor ratio, or ranking of production platforms by cost.

## Figures and Tables

**Table 1:** Direct nucleotide sugar stoichiometry for the synthesis of representative HMOs from lactose.

Target HMO	UDP-GlcNAc	UDP-Gal	GDP-Fuc	CMP-Neu5Ac
2′-FL/3-FL	0	0	1	0
LNT/LNnT	1	1	0	0
LNFP I	1	1	1	0
3′-SL/6′-SL	0	0	0	1
LST a/LST c	1	1	0	1

Values indicate moles of donor directly transferred per mole of target HMO from supplied lactose in canonical sequential GT routes [14,18,19,20]. The table excludes upstream donor formation, nucleotide regeneration, biomass, maintenance, transport, and de novo lactose synthesis. Under the NeuC/NeuB/NeuA route, each CMP-Neu5Ac also requires one upstream UDP-GlcNAc; thus LST a/c requires two UDP-GlcNAc formation events overall, although only one UDP-GlcNAc is used directly in glycan extension. These numbers are neither intracellular concentration ratios nor total cellular ATP costs. 2′-FL, 2′-fucosyllactose; 3-FL, 3-fucosyllactose; LNT, lacto-*N*-tetraose; LNnT, lacto-*N*-neotetraose; LNFP I, lacto-*N*-fucopentaose I; 3′-SL and 6′-SL, 3′- and 6′-sialyllactose; LST a/c, sialyllacto-*N*-tetraose a/c. Donor abbreviations are defined in Section 2.1, Section 2.2, Section 2.3 and Section 2.4.

**Table 2:** Proposed experimental tests for distinguishing candidate limitations.

Candidate	Suggestive Observation	Discriminating Experiment	Interpretive Limit
Analytical validity	Unexpectedly low or variable donor pool	Test quench delay, recovery, leakage, matrix effects and isomer resolution	Only interpret biological differences after measurement performance is adequate.
Donor formation	Low donor with low product rate	Vary donor-forming capacity while holding GT and acceptor conditions as constant as possible; measure active GT and product rate	A higher rate supports a supply contribution; a low pool alone does not.
GT capacity or stability	Donor accumulates while product rate falls	Vary GT dosage or stability independently; measure residual activity and product inhibition	A response with maintained donor formation supports a catalytic constraint; protein amount alone is insufficient.
Acceptor access	Donor and active GT are present but extension is slow	Titrate accessible acceptor; measure intracellular and extracellular acceptor; control for uptake and catabolism	External acceptor addition cannot distinguish transport from intracellular availability without paired measurements.
Transport	Intracellular/extracellular product or intermediate distributions diverge	Perturb a candidate transporter; compare intact and controlled permeabilized cells, viability and leakage	An extracellular titer increase alone can reflect release or lysis rather than synthesis.
Competing metabolism	Extra donor synthesis does not reach target product	Perturb a nonessential candidate sink; trace labeled precursor into target and alternative products	Improved titer may also reflect growth or burden changes; quantify the destination of material.

**Table 3:** Representative outcomes of donor-related engineering and the scope of the supporting evidence.

Study and System	Donor-Related Strategy	Reported Outcome	Interpretation
Dong et al., 2019 *B. subtilis*/LNnT [13]	Combination of two UDP-sugar supply modules	Shake flasks: 1.31 → 1.95 g/L	Supports coordinated donor supply; no universal concentration ratio established
Li et al., 2022 *E. coli*/LNT [15]	Pathway optimization and UTP regeneration	Shake flasks/Fed-batch: 6.16 g/L/57.5 g/L	Combined engineering endpoint; the independent UTP contribution is unresolved
Schelch et al., 2023 Permeabilized cells/3′-SL [17]	Matching donor activation and transferase activities	Whole-cell biocatalysis: 40 ± 4 g/L	Kinetic and intermediate data support catalytic matching; supplied CTP was stoichiometric in this characterization, so the result does not demonstrate an economical CTP-recycling process
Lv et al., 2024 *E. coli*/3′-SL [16]	CTP regeneration with pathway and transport engineering	5 L bioreactor: 56.8 g/L	Nucleotide resources act together with other engineering interventions
Tao et al., 2024 *E. coli*/LNnT [32]	Modification of the LgtA acceptor-binding region	Shake flasks: 1.02 g/L	Product profiles also depend on enzyme selectivity
Hoang et al., 2026 Cell-free enzyme system [21]	UDP-GlcNAc and UDP-Gal synthesis and regeneration	LNnT/LNTII titer (24 h): 1 mM/10mM	Donor platforms can support glycan assembly; resource inputs remain necessary
Cao et al., 2026 Cell-free system/2′-FL [22]	GDP-Fuc pathway with cofactor regeneration	One-pot enzyme reaction: 13.18 mM	An in vitro result requiring separate evaluation from fermentation data

## Data Availability

No new data were created or analyzed in this study. Data sharing is not applicable to this article.

## Supplementary Materials

The following supporting information can be downloaded at: https://www.mdpi.com/article/10.3390/foods15213851/s1, Table S1: Route specific resource accounting; Table S2: Measurements that discriminate pools from flux; Table S3: Inputs for a prospective process cost comparison.
