# Supplementary material for: Connecting Chassis Metabolism with Glycosyltransferase Catalysis: The Role of Nucleotide Sugars in Human Milk Oligosaccharide Biosynthesis
Source: Foods. 2026 Oct 28;15(21):3851. doi: 10.3390/foods15213851 (PMC13763509; doi:10.3390/foods15213851)
Supplement: Supplementary file 1 [file foods-15-03851-s001.zip › foods-4610322-supplementary.pdf]

Supplementary Materials

Connecting Chassis Metabolism with Glycosyltransferase Catalysis: The Role of Nucleotide Sugars in Human Milk Oligosaccharide Biosynthesis

Manuscript ID foods-4610322

These materials document the targeted narrative update and provide proposed measurement and process-accounting tools. They contain no new experimental results. Reference numbers refer to the revised main manuscript.

S1 Targeted literature update and interpretation boundaries

Update date: 15 October 2026. The existing 38-reference bibliography served as the starting set. Titles and DOIs were checked against publisher records and PubMed-indexed records, followed by targeted searches covering HMO production, nucleotide-sugar regeneration, isotope tracing, cell-free and whole-cell systems, microbial quenching and extraction, and pathway thermodynamics. Because this was a targeted narrative update rather than a systematic database search, no PRISMA screening counts are reported.

Purpose	Query or retrieval approach
Production and regeneration	site:pubmed.ncbi.nlm.nih.gov ("human milk oligosaccharides" "nucleotide" "regeneration")
Tracing and turnover	site:pubmed.ncbi.nlm.nih.gov ("nucleotide sugar" "isotope" "metabolism")
Process comparison	site:pubmed.ncbi.nlm.nih.gov ("human milk oligosaccharides" "cell-free" "whole-cell")
Sampling validity	site:pubmed.ncbi.nlm.nih.gov ("metabolomics" "quenching" "extraction" "bacteria")
Thermodynamic interpretation	Noor 2014 pathway thermodynamics kinetic obstacles metabolic pathways PLoS
Record verification	Exact title or DOI for each original reference; targeted searches of publisher records for publication year, volume and article identity.

Studies were selected for direct relevance to Leloir-type HMO synthesis, donor formation or regeneration, donor–catalysis coupling, host constraints, and measurement or mechanistic evidence. Original HMO studies support production claims; broader thermodynamic, burden, and analytical studies are used only for the specific methodological principles they address. Studies on transglycosylation, purely chemical synthesis, or nutrition without a direct production link were excluded from the main synthesis. References [23](Noor et al., 2014) and [35](Bolten et al., 2007) were added for thermodynamic interpretation and sampling-related measurement bias.

Terminology note: In the main text, donor pool refers to the measured amount of a nucleotide sugar at a defined time and normalization basis; donor availability refers to the fraction accessible to the relevant enzyme; donor formation is the rate of synthesis or activation; donor turnover includes target transfer, competing reactions, and degradation; biosynthetic flux is material conversion per unit time; and productive capacity is the condition-dependent ability to sustain target-product formation. These terms are not interchangeable.

Table S1 Route specific resource accounting

Amounts are per mole of HMO from supplied lactose. These are counts of specified pathway events under canonical Glm, GalU/GalE, de novo GDP-Fuc, and NeuC/NeuB/NeuA routes, not total ATP equivalents or experimentally measured fluxes. Salvage or alternative routes change the entries.

Product	Direct donor transfers	Upstream UTP GTP CTP activation events	Additional selected precursor inputs
2′-FL / 3-FL	1 GDP-Fuc	0 / 1 / 0	De novo GDP-Fuc: one hexose precursor and net reducing-equivalent demand; salvage instead adds fucose and ATP-dependent phosphorylation.
LNT / LNnT	1 UDP-GlcNAc + 1 UDP-Gal	2 / 0 / 0	One glutamine-dependent amino-group transfer and one acetyl-CoA-dependent acetylation.
LNFP I	LNT-type donors + 1 GDP-Fuc	2 / 1 / 0	LNT-type inputs plus de novo fucose-route carbon and reducing equivalent.
3′-SL / 6′-SL	1 CMP-Neu5Ac	1 / 0 / 1	One upstream UDP-GlcNAc formation event, one glutamine/acetyl input and one PEP.
LST a / LST c	1 UDP-GlcNAc + 1 UDP-Gal + 1 CMP-Neu5Ac	3 / 0 / 1	Two upstream UDP-GlcNAc formation events in total, two glutamine/acetyl inputs and one PEP.

The first UTP in the NeuC route activates the upstream amino sugar; it is not a direct GT donor transfer in the sialylation step. Recycling UDP/GDP to UTP/GTP requires one phosphorylation, whereas CMP-to-CTP requires two. The ledger excludes carbon-source phosphorylation, nucleotide de novo synthesis, cofactor makeup, biomass, maintenance, transport, by-products, and process losses. Those terms must be added using the actual host and regeneration architecture. Sources: main-text Sections 2.1–2.5 and references(Cao et al., 2025; Chin et al., 2016; Choi et al., 2019; Deng et al., 2019; Dong et al., 2019; Hoang et al., 2026; Hu et al., 2022; Khan et al., 2020; Li et al., 2022; Lv et al., 2024; Schelch et al., 2023).

Table S2 Measurements that discriminate pools from flux

Approach	Information obtained	Essential controls	Limitation
Time-course donor analysis	Follow donor, nucleotides, intermediates and product through growth, induction and production	Validated quenching, recovery, matrix effects, isomer resolution and biomass normalization	Pool trajectories alone do not uniquely determine formation and consumption rates (Bennett et al., 2009; Bolten et al., 2007; Pabst et al., 2010; Rahm et al., 2024).
Isotope tracing	Track label into donor sugar moiety, nucleotide moiety and HMO	Tracer uptake, unlabeled inputs, isotope corrections, pool sizes and sampling times	Enrichment is not an absolute flux; nucleotide labeling can confound sugar-pathway inference(Conte et al., 2023).
Flux modeling	Fit substrate uptake, secretion and labeling to a declared network	Mass balances, biomass composition, compartment assumptions and parameter uncertainty	Steady-state assumptions may fail during induction/feed changes; underdetermined networks yield nonunique fluxes.
GT and donor-enzyme activity	Compare active capacity and residual activity over reaction time	Matched donor/acceptor, pH, temperature and nucleotide-product conditions	Extraction can remove inhibition or localization; soluble protein abundance is not active enzyme(Davidi et al., 2016; Schelch et al., 2023).
Independent perturbations	Vary donor, GT, acceptor access or candidate sink separately; test factorial combinations	Matched growth phase, expression burden, viability and appropriate controls	Compensatory physiology and pleiotropic effects can confound causality(Ceroni et al., 2015; Ceroni et al., 2018; Dong et al., 2019).
Transport and leakage balance	Measure intra-/extracellular intermediates, HMO and quench fractions	Cell viability, lysis and uptake controls; medium carryover correction	Release into broth does not necessarily represent increased synthesis(Bolten et al., 2007; Schelch et al., 2023; Yang et al., 2026).

Table S3 Inputs for a prospective process cost comparison

Use the same target structure, purity specification, scale, accounting period, and price year for each platform. No industrial cost data or techno-economic simulation are reported here. The following variables define what would be required for a valid comparison.

Input	Required quantity or metric	Why it matters
Substrates and regeneration	Mass and purchase cost of all carbon substrates, lactose, salvage sugars, phosphate/energy donors, nucleotide/cofactor makeup	Include feedstocks that are absent from an endpoint yield calculation.
Catalyst preparation	Fermentation, harvest, permeabilization/lysis, purification and immobilization cost per catalyst batch	Cell-free conversion does not eliminate the upstream cost of producing enzymes.
Catalyst life	Cumulative on-spec product per catalyst mass across reuse cycles, with measured activity decline	Initial activity and one successful reuse do not establish lifetime productivity.
Throughput	Reaction and total cycle time, working volume, titer and isolated productivity	Catalyst preparation, cleaning and turnaround alter facility throughput.
Recovery and quality	Isolated recovery, isomer selectivity, impurity clearance and rejected fraction	Only specification-compliant product belongs in the economic denominator.
Scale and utilities	Aeration, agitation, temperature control, water, salts, filtration and waste loads	Benefits seen at laboratory scale may be lost through mass transfer or downstream bottlenecks.

Illustrative calculation: at fixed upstream batch cost and fixed broth target mass, changing isolated recovery from 0.50 to 0.80 multiplies the upstream cost per isolated mass by 0.625. This is not a measured cost reduction and excludes any increased downstream expenditure. Unit conversion only: 13.18 mmol/L × 488.44 mg/mmol = 6437.64 mg/L = 6.44 g/L for anhydrous 2′-FL(Cao et al., 2025). This conversion does not establish isolated yield or comparable process economics.

References

Bennett, B. D., Kimball, E. H., Gao, M., Osterhout, R., Van Dien, S. J., & Rabinowitz, J. D. (2009). Absolute metabolite concentrations and implied enzyme active site occupancy in *Escherichia coli*. *Nature Chemical Biology*, *5*(8), 593–599. https://doi.org/10.1038/nchembio.186

Bolten, C. J., Kiefer, P., Letisse, F., Portais, J.-C., & Wittmann, C. (2007). Sampling for metabolome analysis of microorganisms. *Analytical Chemistry*, *79*(10), 3843–3849. https://doi.org/10.1021/ac0623888

Cao, C., Bai, Y., Zhang, M., Khan, S. S., Ji, Y., & Liu, L. (2025). Synthesis of 2′-fucosyllactose using multi-enzyme cascade with cofactor regeneration. *Scientific Reports*, *16*(1), 2457. https://doi.org/10.1038/s41598-025-32265-7

Ceroni, F., Algar, R., Stan, G.-B., & Ellis, T. (2015). Quantifying cellular capacity identifies gene expression designs with reduced burden. *Nature Methods*, *12*(5), 415–418. https://doi.org/10.1038/nmeth.3339

Ceroni, F., Boo, A., Furini, S., Gorochowski, T. E., Borkowski, O., Ladak, Y. N., Awan, A. R., Gilbert, C., Stan, G.-B., & Ellis, T. (2018). Burden-driven feedback control of gene expression. *Nature Methods*, *15*(5), 387–393. https://doi.org/10.1038/nmeth.4635

Chin, Y.W., Seo, N., Kim, J.H., & Seo, J.H. (2016). Metabolic engineering of *Escherichia coli* to produce 2′-fucosyllactose via salvage pathway of guanosine 5′-diphosphate (GDP)-l-fucose. *Biotechnology and Bioengineering*, *113*(11), 2443–2452. https://doi.org/https://doi.org/10.1002/bit.26015

Choi, Y.H., Park, B.S., Seo, J.H., & Kim, B.G. (2019). Biosynthesis of the human milk oligosaccharide 3-fucosyllactose in metabolically engineered *Escherichia coli* via the salvage pathway through increasing GTP synthesis and β-galactosidase modification. *Biotechnology and Bioengineering*, *116*(12), 3324–3332. https://doi.org/https://doi.org/10.1002/bit.27160

Conte, F., Noga, M. J., van Scherpenzeel, M., Veizaj, R., Scharn, R., Sam, J.E., Palumbo, C., van den Brandt, F. C. A., Freund, C., Soares, E., Zhou, H., & Lefeber, D. J. (2023). Isotopic tracing of nucleotide sugar metabolism in human pluripotent stem cells. *Cells*, *12*(13), 1765.

Davidi, D., Noor, E., Liebermeister, W., Bar-Even, A., Flamholz, A., Tummler, K., Barenholz, U., Goldenfeld, M., Shlomi, T., & Milo, R. (2016). Global characterization of *in vivo* enzyme catalytic rates and their correspondence to *in vitro* k_{cat} measurements. *Proceedings of the National Academy of Sciences*, *113*(12), 3401–3406. https://doi.org/doi:10.1073/pnas.1514240113

Deng, J., Gu, L., Chen, T., Huang, H., Yin, X., Lv, X., Liu, Y., Li, N., Liu, Z., Li, J., Du, G., & Liu, L. (2019). Engineering the substrate transport and cofactor regeneration systems for enhancing 2′-fucosyllactose synthesis in *Bacillus subtilis*. *ACS Synthetic Biology*, *8*(10), 2418–2427. https://doi.org/10.1021/acssynbio.9b00314

Dong, X., Li, N., Liu, Z., Lv, X., Li, J., Du, G., Wang, M., & Liu, L. (2019). Modular pathway engineering of key precursor supply pathways for lacto-*N*-neotetraose production in *Bacillus subtilis*. *Biotechnology for Biofuels*, *12*(1), 212. https://doi.org/10.1186/s13068-019-1551-3

Hoang, T.S., Lange, F., Kleeberg, S.B., Thomas, L., Hoang, N.H., Reichl, U., & Rexer, T. F. T. (2026). Multienzyme platform for the synthesis of UDP sugars and human milk oligosaccharides. *ChemBioChem*, *27*(8), e202500716. https://doi.org/https://doi.org/10.1002/cbic.202500716

Hu, M., Li, M., Miao, M., & Zhang, T. (2022). Engineering *Escherichia coli* for the high-titer biosynthesis of lacto-*N*-tetraose. *Journal of Agricultural and Food Chemistry*, *70*(28), 8704–8712. https://doi.org/10.1021/acs.jafc.2c02423

Khan, M. A., Durica-Mitic, S., Göpel, Y., Heermann, R., & Görke, B. (2020). Small RNA-binding protein RapZ mediates cell envelope precursor sensing and signaling in *Escherichia coli*. *The EMBO Journal*, *39*(6), EMBJ2019103848. https://doi.org/10.15252/embj.2019103848

Li, Z., Zhu, Y., Zhang, P., Zhang, W., & Mu, W. (2022). Pathway optimization and uridine 5′-triphosphate regeneration for enhancing lacto-*N*-tetraose biosynthesis in engineered *Escherichia coli*. *Journal of Agricultural and Food Chemistry*, *70*(25), 7727–7735. https://doi.org/10.1021/acs.jafc.2c02426

Lv, X., Chen, X., Liu, Y., Yuan, L., Wu, J., & Yao, J. (2024). Efficient production of 3′-sialyllactose using *Escherichia coli*. *Journal of Agricultural and Food Chemistry*, *72*(49), 27314–27325. https://doi.org/10.1021/acs.jafc.4c08703

Noor, E., Bar-Even, A., Flamholz, A., Reznik, E., Liebermeister, W., & Milo, R. (2014). Pathway thermodynamics highlights kinetic obstacles in central metabolism. *PLOS Computational Biology*, *10*(2), e1003483. https://doi.org/10.1371/journal.pcbi.1003483

Pabst, M., Grass, J., Fischl, R., Léonard, R., Jin, C., Hinterkörner, G., Borth, N., & Altmann, F. (2010). Nucleotide and nucleotide sugar analysis by liquid chromatography-electrospray ionization-mass spectrometry on surface-conditioned porous graphitic carbon. *Analytical Chemistry*, *82*(23), 9782–9788. https://doi.org/10.1021/ac101975k

Rahm, M., Kwast, H., Wessels, H. J. C. T., Noga, M. J., & Lefeber, D. J. (2024). Mixed-phase weak anion-exchange/reversed-phase LC–MS/MS for analysis of nucleotide sugars in human fibroblasts. *Analytical and Bioanalytical Chemistry*, *416*(15), 3595–3604. https://doi.org/10.1007/s00216-024-05313-w

Schelch, S., Eibinger, M., Zuson, J., Kuballa, J., & Nidetzky, B. (2023). Modular bioengineering of whole-cell catalysis for sialo-oligosaccharide production: coordinated co-expression of CMP-sialic acid synthetase and sialyltransferase. *Microbial Cell Factories*, *22*(1), 241. https://doi.org/10.1186/s12934-023-02249-1

Yang, L., Xu, F., Liu, D., Zhao, C., Qian, Q., Zhu, Y., Ni, D., & Mu, W. (2026). Modular systems engineering enables streamlined development of *Escherichia coli* MG1655 strains for neutral core human milk oligosaccharide synthesis. *Journal of Agricultural and Food Chemistry*, *74*(20), 15706–15714. https://doi.org/10.1021/acs.jafc.6c02888
